# Is *FTO* gene variant related to cancer risk independently of adiposity? An updated meta-analysis of 129,467 cases and 290,633 controls

**DOI:** 10.18632/oncotarget.16446

**Published:** 2017-03-22

**Authors:** Yu Kang, Fang Liu, Yao Liu

**Affiliations:** ^1^ Department of Pharmacy, Southwest Hospital, Third Military Medical University, Chongqing, 400038, China; ^2^ Department of Oncology, Southwest Hospital, Third Military Medical University, Chongqing, 400038, China

**Keywords:** *FTO*, meta-analysis, cancer, obesity

## Abstract

Previous studies have examined the association between the fat mass and obesity-associated (*FTO*) gene variant and risk of cancer in diverse populations. However, the results have been inconsistent. PubMed and Embase databases were searched for the eligible publications in English language by July, 2016. The associations of *FTO* variants with cancer risk were estimated by calculating the pooled odds ratios and 95% confidence intervals by meta-analyses. A total of 27 publications (129,467 cancer cases and 290,633 normal controls) were included in our meta-analysis. Overall, *FTO* rs9939609 variant (or its proxy) was not associated with cancer risk without adjustment for body mass index, as well as additional adjustment for body mss index. However, *FTO* rs9939609 variant was associated with some types of cancer in the subgroup analysis. In addition, overall, there was no significant association between *FTO* rs1477196 variant and cancer risk regardless of adjustment for body mass index. However, *FTO* rs11075995 variant risk allele was associated with breast cancer risk without adjustment for body mass index, but the association disappeared with further adjustment for body mass index. This study overall does not support that the *FTO* variant is associated with cancer risk independently of the adiposity.

## INTRODUCTION

In 2007, the fat mass and obesity associated (*FTO*) gene was reported as the first obesity related gene by the genome-wide association studies (GWAS) in Caucasian population [1, 2]. Subsequently, the following studies confirmed the positive associations between single nucleotide polymorphisms (SNPs) in/near *FTO* gene and obesity risk in diverse populations [3–5].

*FTO* gene was found to affect the function of the central nervous system, as well as adipose tissue at a peripheral level. As obesity is a well established risk factor for most types of cancer, it is interesting and important to investigate whether *FTO* SNPs are associated with risk of cancer. Up to now, a total of 27 publications have examined the associations between *FTO* SNPs and risk of cancer [6–32]. However, the results have been inconsistent. Three meta-analyses have summarized the associations between *FTO* SNPs and risk of cancer [33–35]; however, there are several limitations for them. First, they did not address whether the associations were mediated through body mass index (BMI)/obesity. Second, many eligible studies were omitted. Third, two of three from the same study team examined the association between each of two SNPs (rs8050136[34] and rs9939609[35]) in/near *FTO* gene and cancer risk. It is illogical to do the separate analyses for these two SNPs as they are in strong linkage disequilibrium (LD, *r*^2^>0.90) in both European and Asian populations.

Therefore, we aimed to perform an updated meta-analysis to investigate the associations between *FTO* rs9939609 SNP (or any proxy SNP, *r*^2^>0.90) and other SNPs which are not in tight LD with rs9939609 SNP (such as rs1477196 and rs11075995) and cancer risk. In addition, we also aimed to examine whether the associations are independent of adiposity.

## RESULTS

### Characteristics of the studies

**Figure 1:**
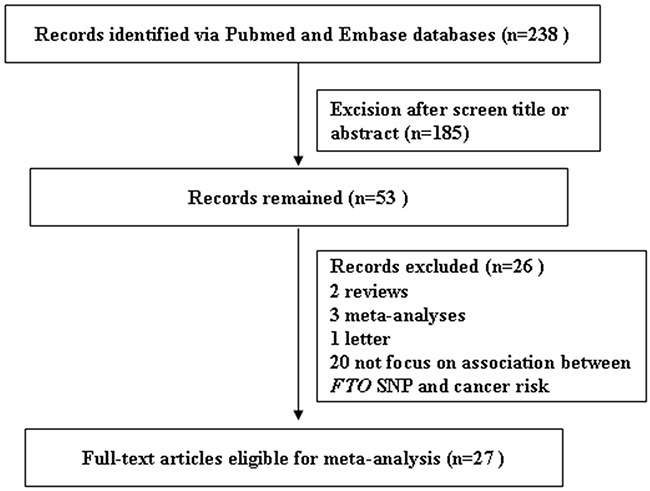
Flowchart for inclusion/exclusion of studies

A flow chart describing the process of inclusion/exclusion of studies is presented in Figure 1. The literature search identified a total of 238 potentially relevant articles. At last, a total of 27 publications (129,467 cancer cases and 290,633 normal controls) were included in our meta-analysis. There were 24 publications (113780 cases and 210593 controls) for *FTO* rs9939609 SNP, 5 publications (1594 cases and 2034 controls) for *FTO* rs1477196 SNP, and 3 publications (14144 cases and 79973 controls) for rs11075995 variant. All three SNPs in the each of included studies were in Hardy-Weinberg Equivalent. The characteristics of the included studies are listed in Table 1.

**Table 1:** The detailed characteristics of the included studies in the meta-analysis

Study *	Country	Ethnicity	Type ofcancer	No. of cases	No. of controls	OR	95% CI		SNP	Adjustmentfor BMI
Brennan, 2009 [6]	Czech Republic, Hungary, Poland, Romania, Russia, and Slovakia	European	Lung cancer	2250	3052	0.92	0.84	1.00	rs9939609	No
Brennan, 2009 [6]	Czech Republic, Hungary, Poland, Romania, Russia, and Slovakia	European	Kidney cancer	954	3052	1.06	0.95	1.19	rs9939609	No
Brennan, 2009 [6]	Czech Republic, Hungary, Poland, Romania, Russia, and Slovakia	European	Upper aerodigestive cancer	811	3052	0.98	0.87	1.12	rs9939609	No
Gaudet, 2010 [7]	USA and Australia	Mixed	Endometrial cancer	417	406	1.05	0.86	1.28	rs8050136	No
Lewis, 2010 [8]	UK	European	Prostate cancer	1550	1815	0.94	0.85	1.03	rs9939609	Yes
Meyer, 2010 [9]	USA	Mixed	Prostate cancer	379	5874	1.04	0.91	1.20	rs8050136	No
Delahanty, 2011 [10]	China	East Asian	Endometrial cancer	832	2049	1.07	0.89	1.29	rs9939609	No
Kaklamani, 2011 [11]	USA	Mixed	Breast cancer	302	349	0.992	0.78	1.26	rs9939609	No
						0.975	0.77	1.23		Yes
						1.408	1.11	1.79	rs1477196	No
						1.447	1.13	1.85		Yes
Lurie, 2011 [12]	Australia, USA, Poland, and Canada	European	Endometrial cancer	3561	5167	1.07	0.99	1.14	rs9939609	No
						1.01	0.94	1.08		Yes
Pierce, 2011 [13]	Finland, USA, China, France, Germany, Greece, Italy, The Netherlands, Spain, and the UK	European	Pancreatic cancer	1763	1802	1.12	1.02	1.23	rs8050136	No
Tang, 2011 [14]	USA	Mixed	Pancreatic cancer	1053	1130	1.08	0.96	1.22	rs9939609	No
						1.03	0.80	1.30		Yes
Brooks, 2012 [15]	USA and Denmark	European	Breast cancer	643	1271	1.1	0.9	1.3	rs9939609	No
Hubacek, 2012 [16]	Czech Republic, Hungary, Poland, Romania, Russia, and Slovakia	European	Colorectal cancer	1005	6827	1.02	0.93	1.13	rs17817449	No
Kitahara, 2012 [17]	USA	European	Thyroid cancer	341	444	0.77	0.62	0.94	rs9939609	No
						0.76	0.61	0.93		Yes
						1.31	1.07	1.61	s1477196	No
						1.32	1.07	1.61		Yes
Kusinska, 2012 [18]	Poland	European	Breast cancer	134	357	1.05	0.68	1.61	rs9939609	No
Lim, 2012 [19]	USA	Mixed	Colorectal cancer	2033	9640	1.02	0.93	1.11	rs9939609	No
Machiela, 2012 [20]	USA and several European countries	European	Prostate cancer	2782	4458	0.93	0.86	1.00	rs9939609	Yes
Tarabra, 2012 [21]	Italy	European	Colorectal cancer	341	311	1.01	0.81	1.25	rs9939609	No
Akilzhanova, 2013 [22]	Kazakhstan	European	Breast cancer	315	604	0.96	0.78	1.17	rs1477196	No
						0.96	0.78	1.17		Yes
da Cunha, 2013 [23]	Brazil	European	Breast cancer	100	148	0.86	0.60	1.25	rs9939609	No
						0.87	0.61	1.26		Yes
Garcia-Closas, 2013 [24]	USA and many European countries	European	Breast cancer	10706	76647	1.11	1.07	1.15	rs11075995	No
				3071	20130	1.16	1.09	1.24		Yes
Iles, 2013 [25]	European countries	European	Melanoma	13060	60726	1.03	0.97	1.10	rs8050136	No
Lin, 2013 [26]	Japan	East Asian	Pancreatic cancer	360	400	1.33	1.04	1.72	rs9939609	No
						1.41	1.07	1.85		Yes
Long, 2013 [27]	USA	African	Breast cancer	1113	930	1.21	1.06	1.37	rs17817449	Yes
Zheng, 2013 [28]	China, Korea, Japan and Thailand	East Asian	Breast cancer	16797	18983	0.92	0.88	0.97	rs17817449	No
Zhang, 2014 [29]	China	East Asian	Breast cancer	2901	2789	1.06	0.98	1.14	rs11075995	No
Mojaver, 2015 [30]	Iran	Middle East	Breast cancer	99	100	0.85	0.51	1.41	rs9939609	No
						1.215	0.683	2.161		Yes
						1.14	0.64	2.01	rs1477196	No
						0.890	0.464	1.707		Yes
Zeng,2015 [31]	China	East Asian	Breast cancer	537	537	1.19	0.90	1.57	rs9939609	No
						1.18	0.89	1.56		Yes
						0.73	0.58	0.93	rs1477196	No
						0.75	0.59	0.96		Yes
						0.90	0.71	1.15	rs11075995	No
						0.94	0.73	1.20		Yes
Zhao, 2016[32]	Several European countries	European	Breast cancer	62328	83817	0.94	0.92	0.95	rs9939609	No

* All included studies were case-control designed.

### Meta-analysis results

**Figure 2:**
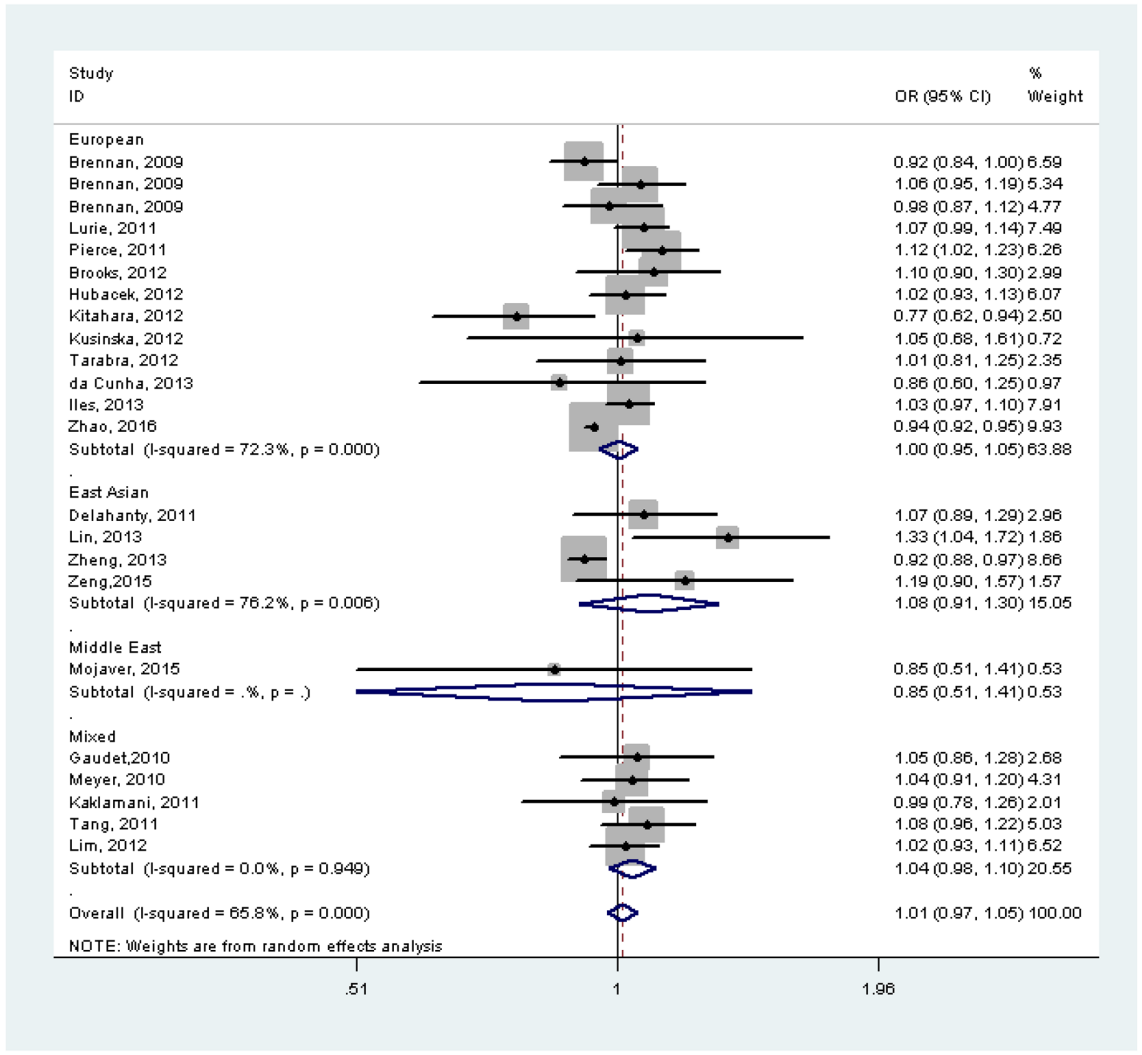
Forest plot of the effect of *FTO* rs9939609 on risk of cancer by race/ethnicity without adjustment for body mass index

**Figure 3:**
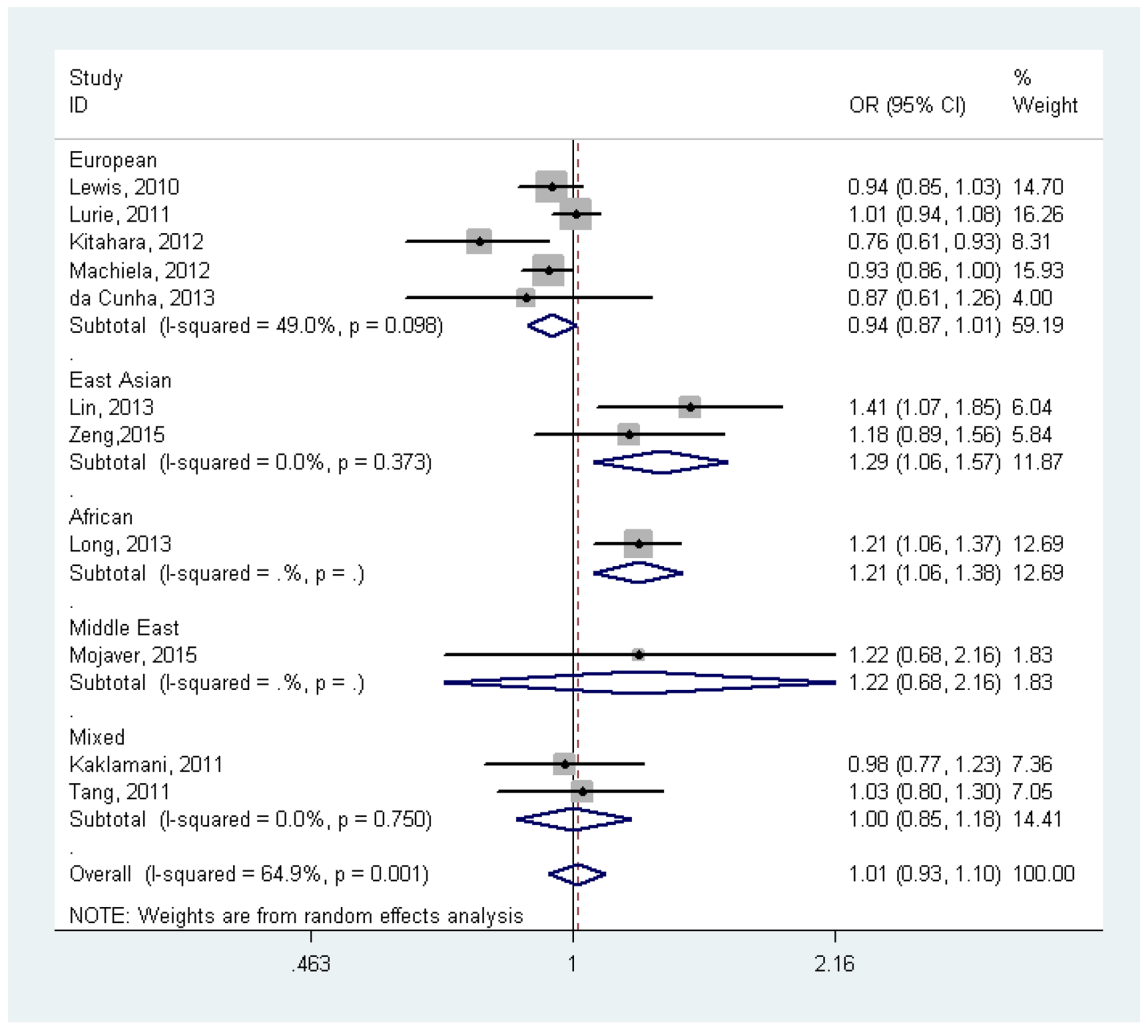
Forest plot of the effect of *FTO* rs9939609 on risk of cancer by race/ethnicity with adjustment for body mass index

Overall, *FTO* rs9939609 SNP was not associated with cancer risk without adjustment for BMI (OR=1.01, 95%CI=0.97-1.05). In the subgroup analysis by race/ethnicity, before adjustment for BMI, there was no any significant associations in European population, East Asian population, Middle East population and mixed population (all *P*>0.05) (Figure 2). After adjustment for BMI, *FTO* rs9939609 SNP risk allele was associated with cancer risk in East Asian population (OR=1.29, 95%CI=1.06-1.57) and African population (OR=1.21, 95%CI=1.06-1.38), but not in European population, Middle East population and Mixed population (all *P*>0.05) (Figure 3). In the subgroup analysis by cancer type, *FTO* rs9939609 SNP risk allele marginally increased risk of endometrial cancer (OR=1.07, 95%CI=1.00-1.14) and pancreatic cancer (OR=1.12, 95%CI=1.04-1.21), while it marginally decreased risk of breast cancer (OR=0.94, 95%CI=0.92-0.96) (Table 2 and Supplementary Figure 1). Overall, there was also no significant association between *FTO* rs9939609 SNP and cancer risk with adjustment for BMI (OR=1.01, 95%CI=0.93-1.10). *FTO* rs9939609 SNP risk allele marginally decreased risk of prostate cancer (OR=0.93, 95%CI=0.88-0.99), while it marginally increased risk of breast cancer (OR=1.12, 95%CI=0.99-1.26) (Table 2 and Supplementary Figure 2).

**Table 2:** Associations between *FTO* variants and cancer risk by cancer type

	OR	95% CI	*I*^2^ (%)	*P*_for heterogeneity_
**rs9939609**				
Before BMI adjustment				
All	1.01	0.97-1.05	65.8	<0.001
Endometrial cancer	1.07	1.00-1.14	0	0.985
Breast cancer	0.94	0.92-0.96	1.8	0.416
Pancreatic cancer	1.12	1.04-1.21	6.8	0.342
Colorectal cancer	1.02	0.96-1.09	0	0.996
Others	0.98	0.92-1.05	57.4	0.038
After BMI adjustment				
All	1.01	0.93-1.10	64.9	0.001
Breast cancer	1.12	0.99-1.26	14.2	0.324
Pancreatic cancer	1.20	0.88-1.63	64.7	0.093
Prostate cancer	0.93	0.88-0.99	0	0.864
Others	0.89	0.68-1.18	84.1	0.012
**rs1477196**				
Before BMI adjustment				
All	1.07	0.97-1.20	80.1	<0.001
Breast cancer	1.00	0.88-1.13	80.2	0.002
Thyroid cancer	1.31	1.07-1.61	-	-
After BMI adjustment				
All	1.08	0.97-1.21	79.4	0.001
Breast cancer	1.00	0.88-1.14	79.1	0.002
Thyroid cancer	1.32	1.08-1.62	-	-
**rs11075995**				
Before BMI adjustment				
Breast cancer	1.08	1.01-1.15	47.2	0.150
After BMI adjustment				
Breast cancer	1.08	0.89-1.31	61.2	0.108

There was no significant association between *FTO* rs1477196 SNP and cancer risk without (OR=1.07, 95%CI= 0.97-1.20) or with (OR=1.08, 95%CI=0.97-1.21) adjustment for BMI. However, we found a significant association between *FTO* rs1477196 SNP and risk of thyroid cancer without (OR=1.31, 95%CI=1.07-1.61) or with (OR=1.32, 95%CI=1.08-1.62) adjustment for BMI (Table 2 and Supplementary Figures 3-4).

*FTO* rs11075995 SNP risk allele was associated with breast cancer risk without adjustment for BMI (OR=1.08, 95%CI=1.01-1.15) (Table 2 and Supplementary Figure 5). However, the significant association disappeared after adjustment for BMI (OR=1.08, 95%CI=0.89-1.31) (Table 2 and Supplementary Figure 6).

### Publication bias

There was no publication bias for *FTO* rs9939609, rs1477196 or rs11075995 SNP using Begg's test or Egger's test (all *P*>0.05).

## DISCUSSION

Our updated meta-analysis shows that *FTO* rs9939609 SNP was associated with some types of cancer, such as endometrial cancer, pancreatic cancer and breast cancer without adjustment for BMI, while it was still associated with breast cancer and prostate cancer with adjustment for BMI. In addition, *FTO* rs1477196 SNP was associated with thyroid cancer independently of BMI and *FTO* rs11075995 SNP was associated with breast cancer dependently on BMI.

Several meta-analyses have addressed the association between *FTO* SNP and risk of diabetes, [36] hypertension, [37] cardiovascular disease, [38] polycystic ovary syndrome [39] and mortality [40]. Most of these meta-analyses supported *FTO* SNP was associated with health outcomes independently of adiposity. A meta-analysis of data from 169,551 Caucasian adults showed that the hazards ratio (HR) for the A minor allele of the *FTO* rs9939609 SNP was 1.02 (1.00–1.04, *P*=0.097), but the association disappeared after adjustment for BMI (HR=1.00; 0.98–1.03, *P*=0.662) [40]. These results suggested that *FTO* SNP risk allele increases risk of mortality directly through adiposity pathway.

It seemed that *FTO* rs9939609 SNP played different roles in the development of different cancer, as well as in different populations. Previous studies demonstrated that BMI was associated with risk of common cancer, but its association with some cancer types differed between sexes and different ethnic populations [41]. As *FTO* SNP rs9939609 was strongly associated with BMI, it is not surprising that this variant was associated with some types of cancer but not with other types of cancer.

The FTO protein is highly expressed in hypothalamus, as well as in many other tissues: mesenteric fat, adipose, pancreatic, and liver. It regulates the global metabolic rate, energy expenditure, energy homeostasis, body size and body fat accumulation [42]. *FTO* rs8050136 was reported to preferentially bind to cut-like homeobox (*CUTL*1) in human fibroblast DNA and silencing this transcriptional factor CUTL1 could lead to decreased FTO expression in fibroblasts [43]. In addition, *FTO* SNP was strongly associated with expression of a tumor suppressor/cell cycle-repressing gene, namely retinoblastoma-like 2 [44]. Further studies are necessary to clarify the underlying mechanism between *FTO* SNP and cancer risk.

Our study has several strengths. First, our study included 27 publications consisting of ~130, 000 cases and ~300,000 controls, which had the larger statistical power than three previous meta-analyses [33–35]. Second, we presented results without and with adjustment for BMI, but the previous three meta-analyses didn’t. Third, besides rs9939609 and its proxy SNP (rs8050136 and rs17817449), we also investigated two other SNPs (rs1477196 or rs11075995), which are not in high LD with rs9939609. However, several limitations should be noted. First, the effects of gene-gene/gene-environment interactions were not addressed in this meta-analysis as the included individual studies did not provided us with these data. Second, although the total sample size was large enough, it was still limited for some types of cancer. Thus, the subgroup results with limited statistical power should be interpreted with caution. Third, there was significant heterogeneity between studies for three SNPs and the results should be interpreted cautiously.

In conclusion, our updated meta-analysis supported that *FTO* SNP was associated with some types of cancer, which was mediated by BMI or independent of BMI. Further studies should focus on gene-gene/gene-environment interaction in the development of cancer. Epigenetics and metabonomics should be paid more attention in order to solve how BMI modify the association between *FTO* SNP and cancer risk.

## MATERIALS AND METHODS

### Literature and search strategy

We searched PubMed and Embase databases for the potentially eligible studies. The following key words were used to search the eligible publications: (fat-mass and obesity-associated gene OR *FTO*) and (polymorphism OR variant OR variation OR genotype) and (cancer OR tumor OR carcinoma). We restricted publication language to English. The reference lists of retrieved articles were also hand-searched. The literature search was updated by July 14, 2016.

### Inclusion criteria and data extraction

The included studies met all the following inclusion criteria: (1) investigation of the association of *FTO* rs9939609 SNP (or any proxy SNP (rs8050136, rs17817449), *r*^2^>0.90) or other SNPs which are not in tight LD with rs9939609 (such as rs1477196 and rs11075995) with cancer risk; (2) use of a case–control or cohort design; and (3) provision of an odds ratio (OR) with 95% confidence interval (CI) with or without adjustment for body mass index (BMI). The following information was extracted from each study: (1) name of the first author; (2) year of publication; (3) country of origin; (4) race/ethnicity of the study population; (5) number of cases and controls; (6) type of cancer; (7) studied SNP; and (8) whether adjusted for BMI in the logistical regression model. Two authors independently reviewed the articles for compliance with the inclusion/exclusion criteria, resolved disagreements and reached a consistent decision after discussion with the third author.

### Statistical analysis

The associations of *FTO* SNPs with cancer risk were estimated by calculating the pooled ORs and 95% CIs under an additive genetic model. The significance of the OR was determined by the Z test (*p*<0.05 was considered statistically significant). Cochrane's Q test was performed to test the between-study heterogeneity [45, 46]. *I*^2^ represents the range for degree of heterogeneity. A random-effects (DerSimonian–Laird [45]) or fixed-effects (Mantel–Haenszel [46]) model was used to calculate the pooled OR in the presence (*p*≤0.10 or *I*^2^≥50%) or absence (*p*>0.10 and *I*^2^<50%) of heterogeneity, respectively. Publication bias was assessed by Begg's test and Egger's test [47] (*p*<0.05 was considered statistically significant). Data were analyzed using STATA version 11.0 (StataCorp LP, College Station, TX, USA).

## SUPPLEMENTARY MATERIALS FIGURES


