# Supplementary material for: Is FTO gene variant related to cancer risk independently of adiposity? An updated meta-analysis of 129,467 cases and 290,633 controls
Source: Oncotarget. 2017 Mar 22;8(31):50987–96. doi: 10.18632/oncotarget.16446 (PMC5584223; doi:10.18632/oncotarget.16446)
Supplement: Supplementary file 1 [file oncotarget-08-50987-s001.pdf]

Is *FTO* gene variant related to cancer risk independently of adiposity? An updated meta-analysis of 129,467 cases and 290,633 controls

SUPPLEMENTARY FIGURES

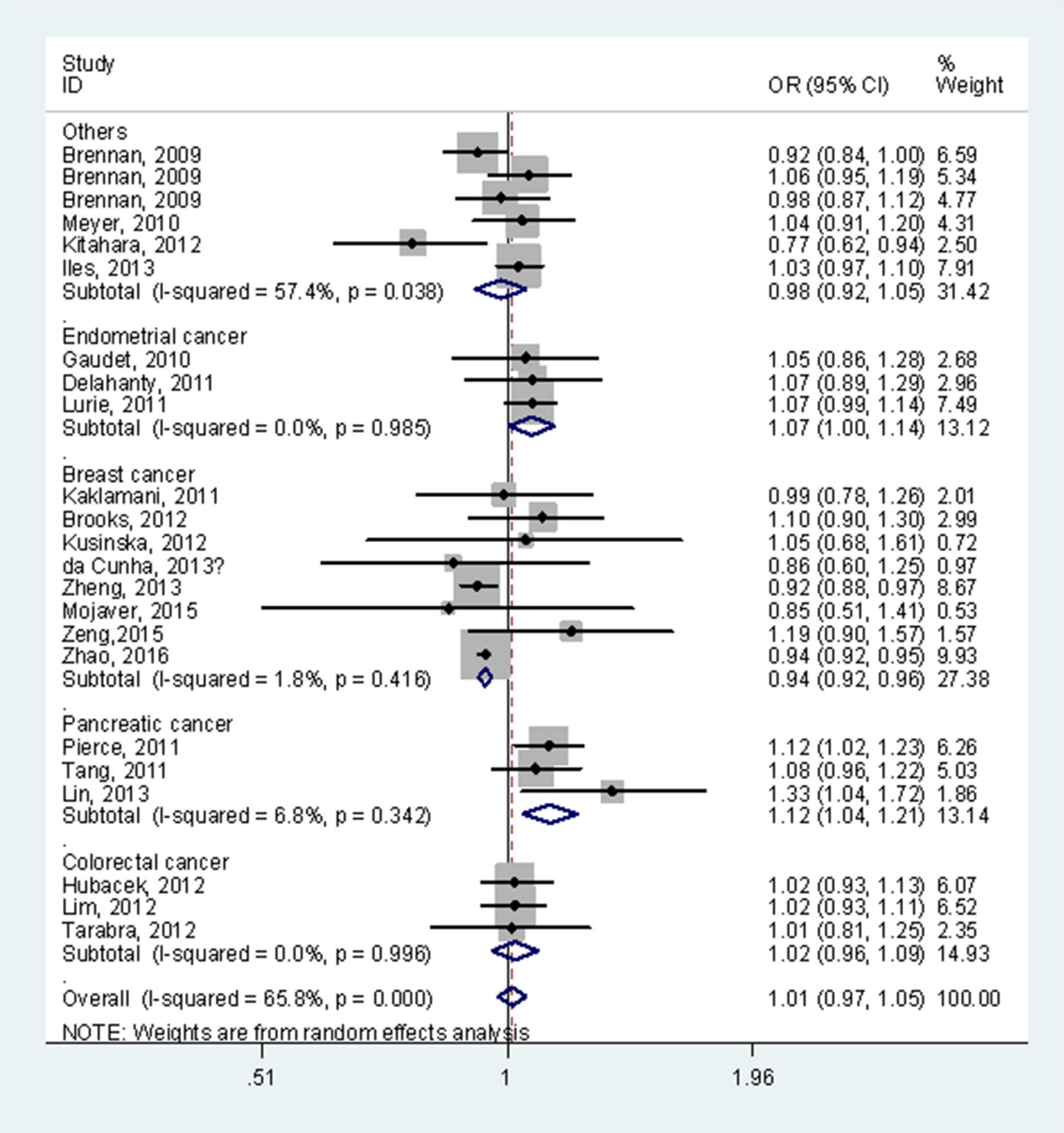

Supplementary Figure 1: Forest plot of the effect of *FTO* rs9939609 on risk of cancer by cancer type without adjusted for body mass index.

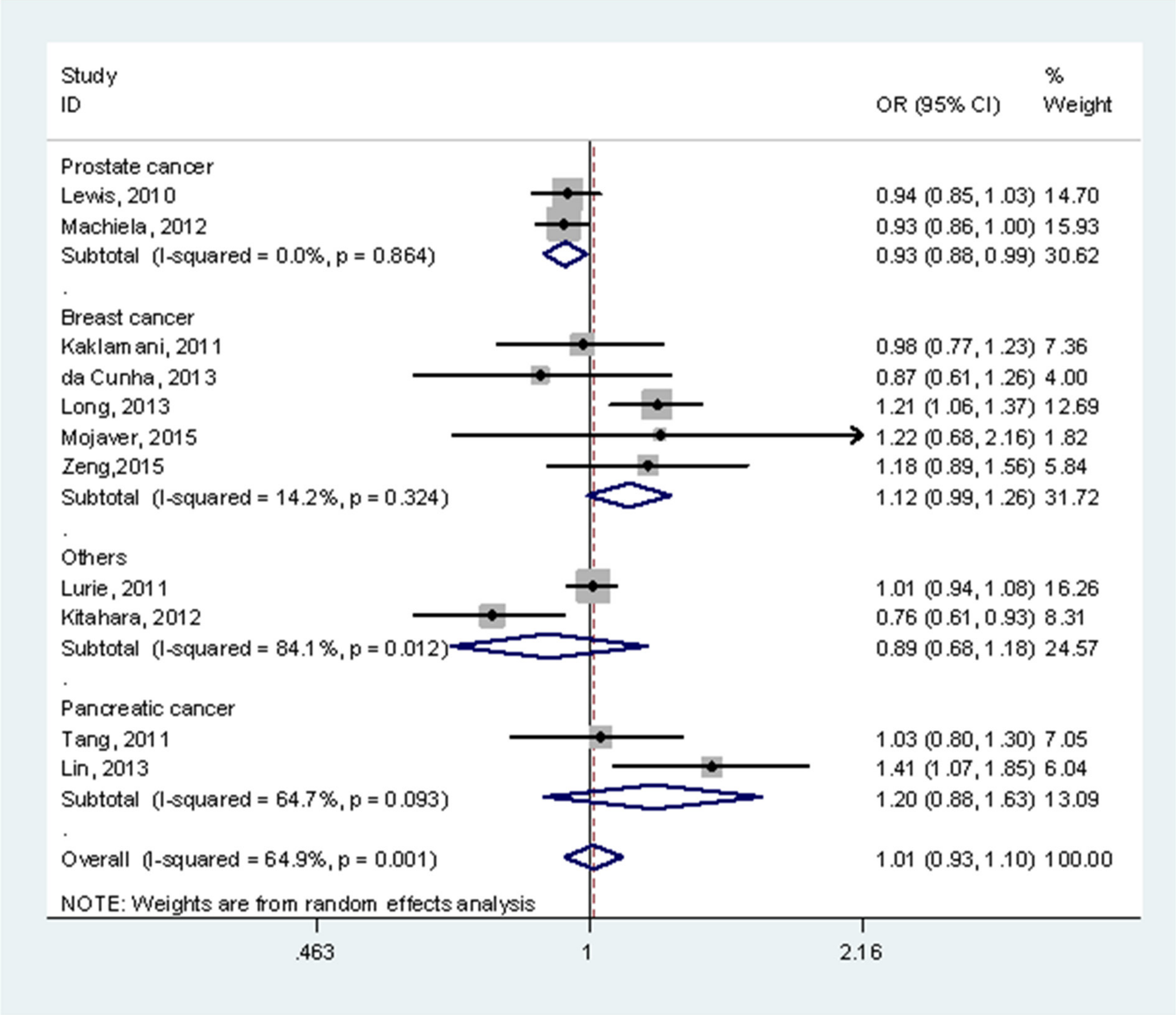

Supplementary Figure 2: Forest plot of the effect of *FTO* rs9939609 on risk of cancer by cancer type with adjusted for body mass index.

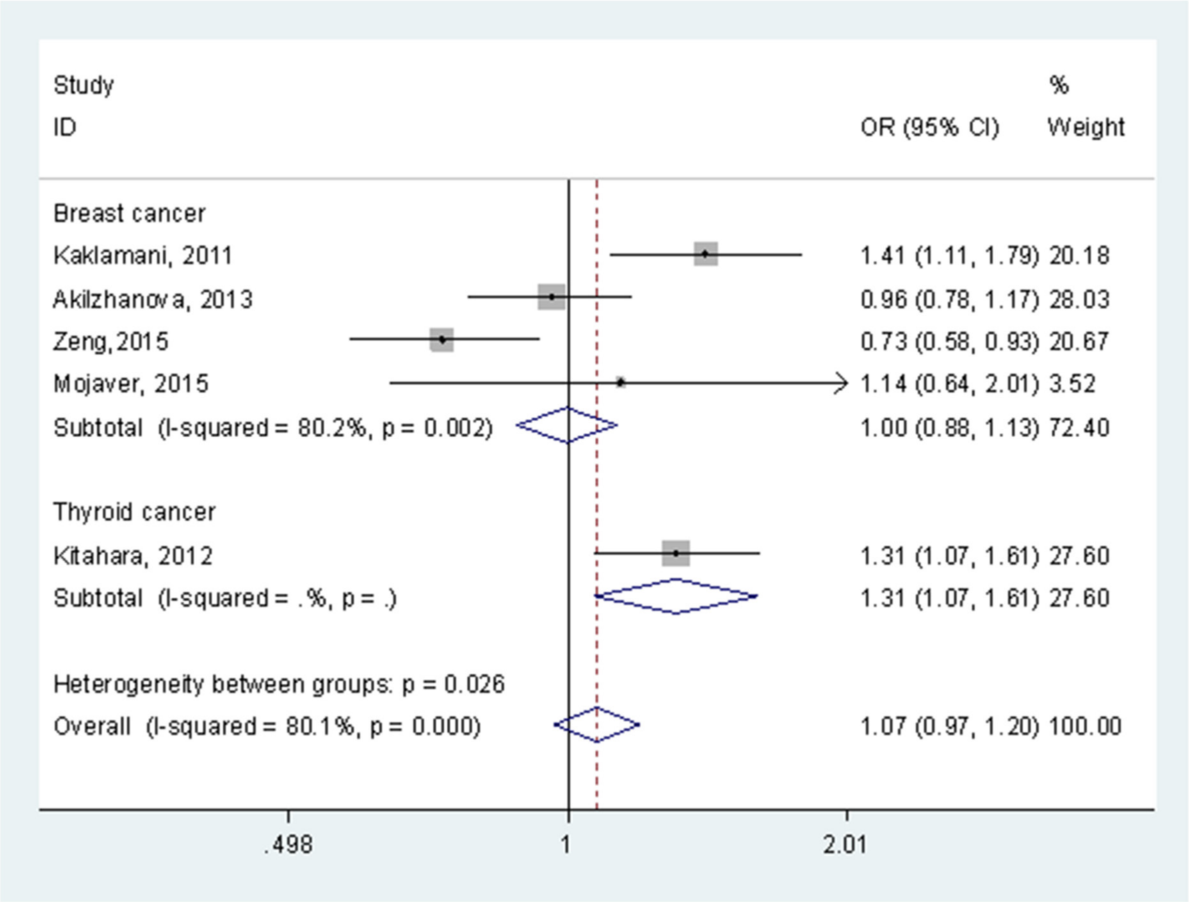

Supplementary Figure 3: Forest plot of the effect of *FTO* rs1477196 on risk of cancer by cancer type without adjusted for body mass index.

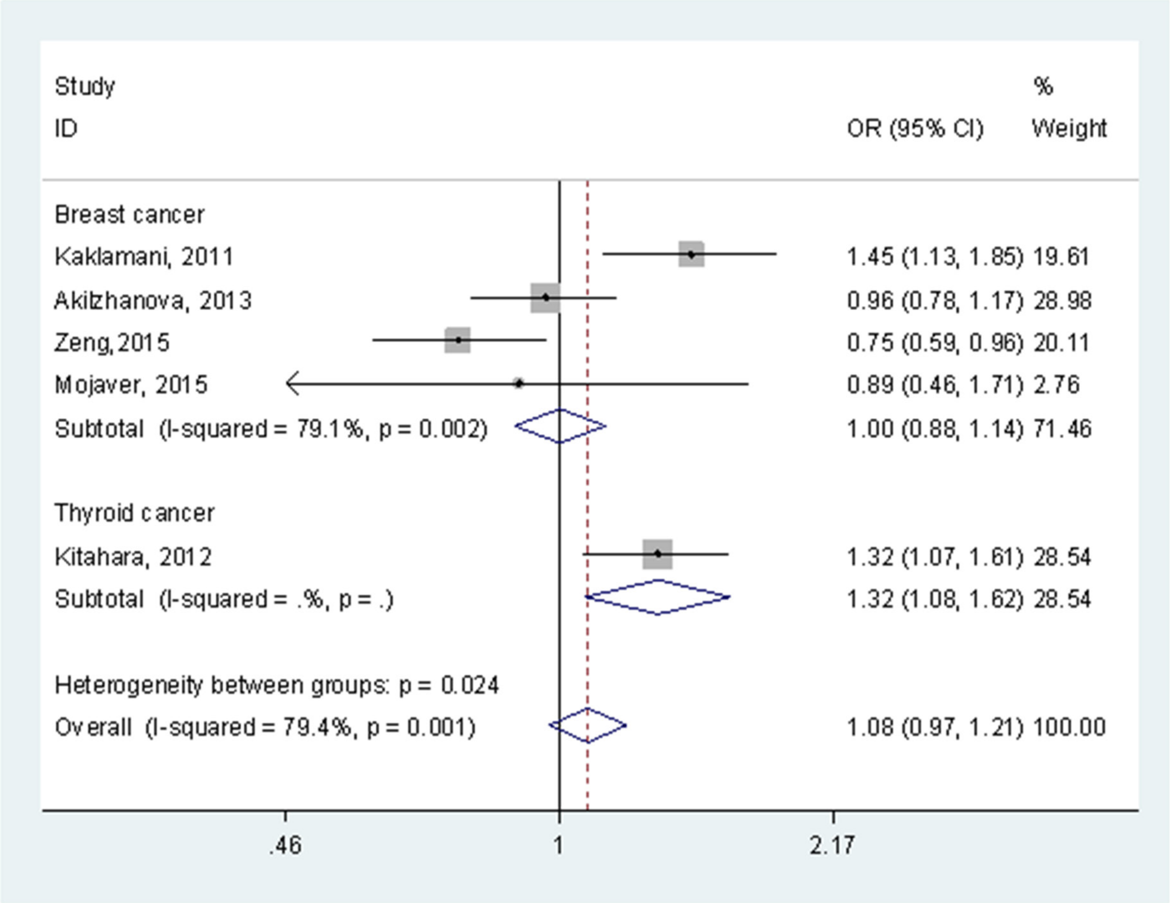

Supplementary Figure 4: Forest plot of the effect of *FTO* rs1477196 on risk of cancer by cancer type with adjusted for body mass index.

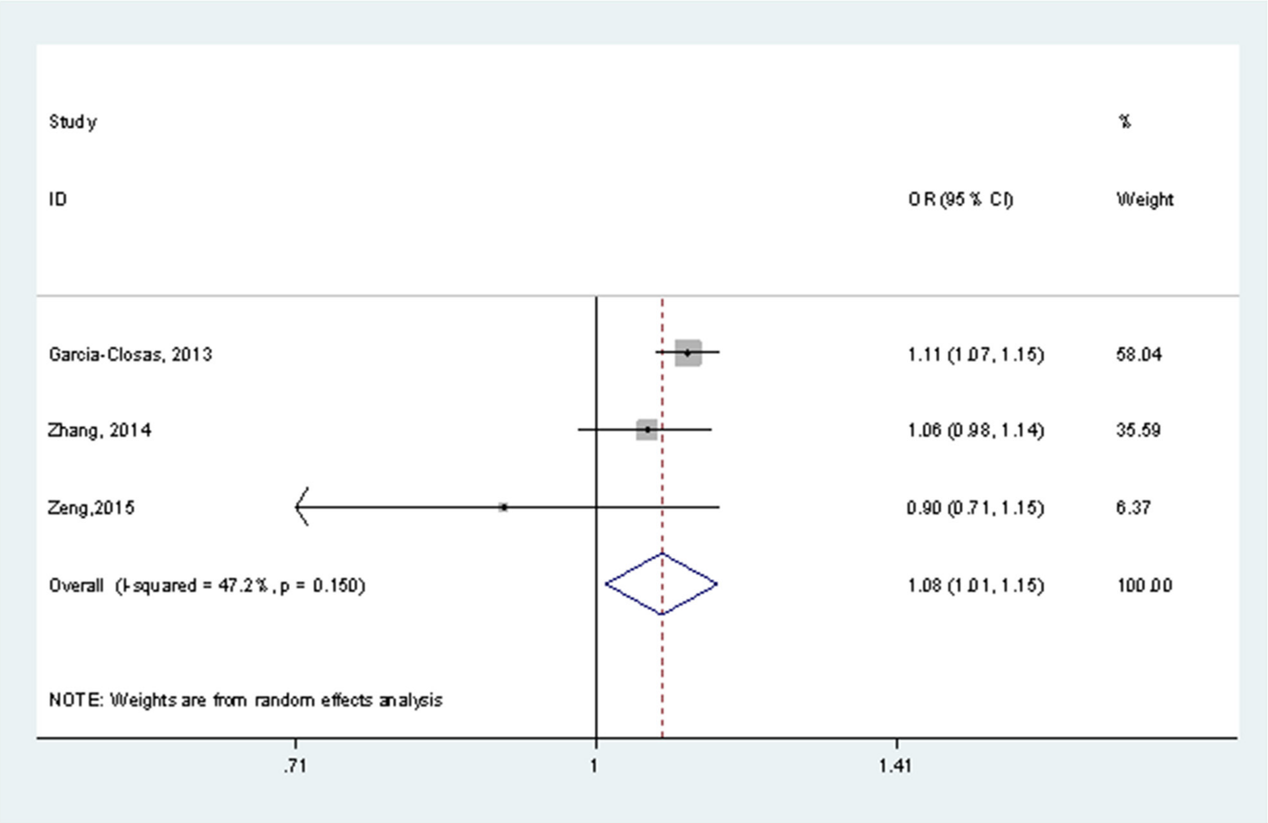

Supplementary Figure 5: Forest plot of the effect of *FTO* rs11075995 on risk of cancer without adjusted for body mass index.

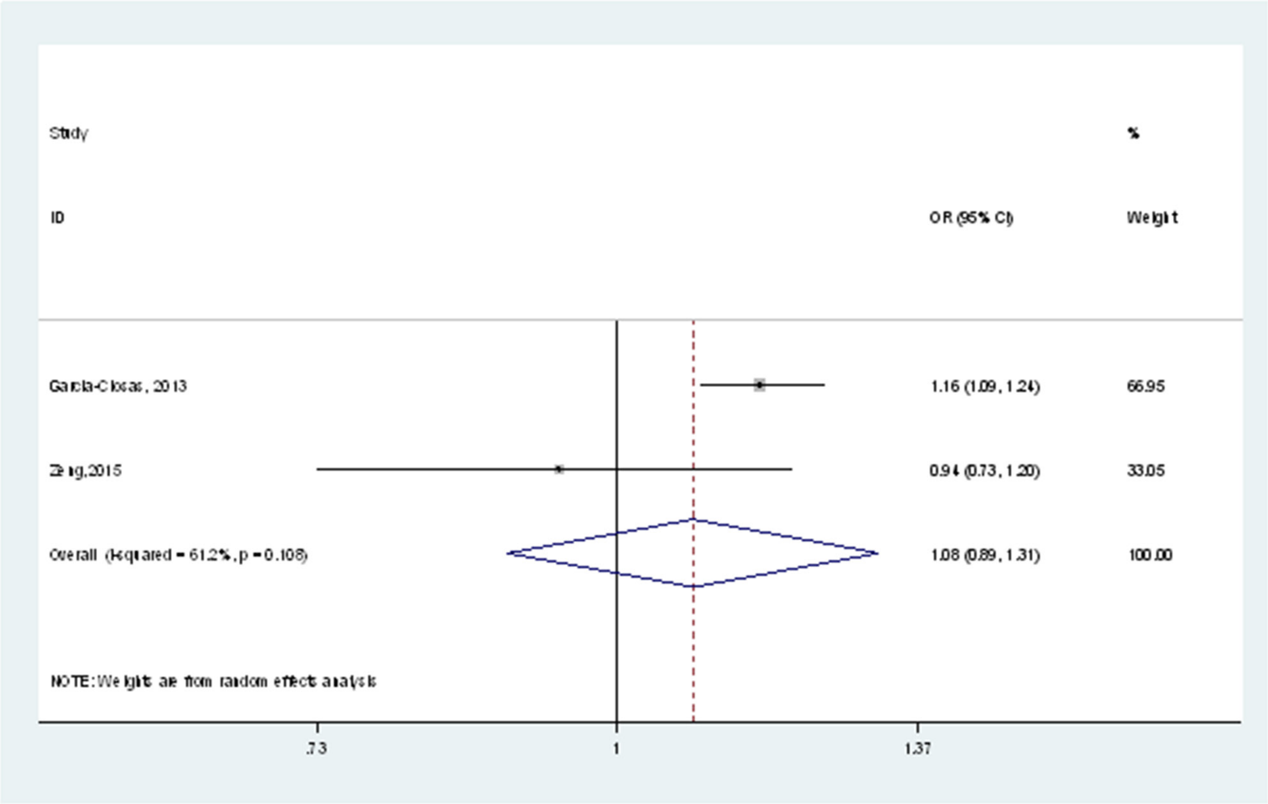

Supplementary Figure 6: Forest plot of the effect of *FTO* rs11075995 on risk of cancer with adjusted for body mass index.
